# Intracardiac echocardiography guided anatomical approach to cardioneuroablation: feasibility and outcomes

**DOI:** 10.1093/europace/euaf295

**Published:** 2025-11-18

**Authors:** Medhat Farwati, Ayman A Hussein, Santiago Giraldo, William Bautista, Koji Higuchi, Bryan Baranowski, Mandeep Bhargava, Thomas D Callahan, Mina K Chung, Roy Chung, Arwa Younis, Jeffery Courson, Thomas J Dresing, Mohamed Kanj, Arshneel Kochar, Robert Koeth, Justin Z Lee, Ioan Liuba, David O Martin, Kenneth Mayuga, Shady Nakhla, John Rickard, Walid I Saliba, Jakub Sroubek, Tyler L Taigen, Niraj Varma, James Witten, Edward Soltesz, Carlos Tapias, Luis Saenz, Oussama Wazni, Pasquale Santangeli

**Affiliations:** Department of Cardiovascular Medicine, Heart, Vascular, and Thoracic Institute, 9500 Euclid Ave., Cleveland Clinic, Cleveland, OH 44195, USA; Department of Cardiovascular Medicine, Heart, Vascular, and Thoracic Institute, 9500 Euclid Ave., Cleveland Clinic, Cleveland, OH 44195, USA; International Arrhythmia Center, Fundación Cardioinfatil - La Cardio, Division of Cardiology, Bogota, Colombia; International Arrhythmia Center, Fundación Cardioinfatil - La Cardio, Division of Cardiology, Bogota, Colombia; Department of Cardiovascular Medicine, Heart, Vascular, and Thoracic Institute, 9500 Euclid Ave., Cleveland Clinic, Cleveland, OH 44195, USA; Department of Cardiovascular Medicine, Heart, Vascular, and Thoracic Institute, 9500 Euclid Ave., Cleveland Clinic, Cleveland, OH 44195, USA; Department of Cardiovascular Medicine, Heart, Vascular, and Thoracic Institute, 9500 Euclid Ave., Cleveland Clinic, Cleveland, OH 44195, USA; Department of Cardiovascular Medicine, Heart, Vascular, and Thoracic Institute, 9500 Euclid Ave., Cleveland Clinic, Cleveland, OH 44195, USA; Department of Cardiovascular Medicine, Heart, Vascular, and Thoracic Institute, 9500 Euclid Ave., Cleveland Clinic, Cleveland, OH 44195, USA; Department of Cardiovascular Medicine, Heart, Vascular, and Thoracic Institute, 9500 Euclid Ave., Cleveland Clinic, Cleveland, OH 44195, USA; Department of Cardiovascular Medicine, Heart, Vascular, and Thoracic Institute, 9500 Euclid Ave., Cleveland Clinic, Cleveland, OH 44195, USA; Department of Cardiovascular Medicine, Heart, Vascular, and Thoracic Institute, 9500 Euclid Ave., Cleveland Clinic, Cleveland, OH 44195, USA; Department of Cardiovascular Medicine, Heart, Vascular, and Thoracic Institute, 9500 Euclid Ave., Cleveland Clinic, Cleveland, OH 44195, USA; Department of Cardiovascular Medicine, Heart, Vascular, and Thoracic Institute, 9500 Euclid Ave., Cleveland Clinic, Cleveland, OH 44195, USA; Department of Cardiovascular Medicine, Heart, Vascular, and Thoracic Institute, 9500 Euclid Ave., Cleveland Clinic, Cleveland, OH 44195, USA; Department of Cardiovascular Medicine, Heart, Vascular, and Thoracic Institute, 9500 Euclid Ave., Cleveland Clinic, Cleveland, OH 44195, USA; Department of Cardiovascular Medicine, Heart, Vascular, and Thoracic Institute, 9500 Euclid Ave., Cleveland Clinic, Cleveland, OH 44195, USA; Department of Cardiovascular Medicine, Heart, Vascular, and Thoracic Institute, 9500 Euclid Ave., Cleveland Clinic, Cleveland, OH 44195, USA; Department of Cardiovascular Medicine, Heart, Vascular, and Thoracic Institute, 9500 Euclid Ave., Cleveland Clinic, Cleveland, OH 44195, USA; Department of Cardiovascular Medicine, Heart, Vascular, and Thoracic Institute, 9500 Euclid Ave., Cleveland Clinic, Cleveland, OH 44195, USA; Department of Cardiovascular Medicine, Heart, Vascular, and Thoracic Institute, 9500 Euclid Ave., Cleveland Clinic, Cleveland, OH 44195, USA; Department of Cardiovascular Medicine, Heart, Vascular, and Thoracic Institute, 9500 Euclid Ave., Cleveland Clinic, Cleveland, OH 44195, USA; Department of Cardiovascular Medicine, Heart, Vascular, and Thoracic Institute, 9500 Euclid Ave., Cleveland Clinic, Cleveland, OH 44195, USA; Department of Cardiovascular Medicine, Heart, Vascular, and Thoracic Institute, 9500 Euclid Ave., Cleveland Clinic, Cleveland, OH 44195, USA; Department of Cardiovascular Medicine, Heart, Vascular, and Thoracic Institute, 9500 Euclid Ave., Cleveland Clinic, Cleveland, OH 44195, USA; Department of Cardiovascular Medicine, Heart, Vascular, and Thoracic Institute, 9500 Euclid Ave., Cleveland Clinic, Cleveland, OH 44195, USA; Department of Cardiac Surgery, Heart, Vascular, and Thoracic Institute, Cleveland Clinic, Cleveland, OH 44195, USA; Department of Cardiac Surgery, Heart, Vascular, and Thoracic Institute, Cleveland Clinic, Cleveland, OH 44195, USA; International Arrhythmia Center, Fundación Cardioinfatil - La Cardio, Division of Cardiology, Bogota, Colombia; International Arrhythmia Center, Fundación Cardioinfatil - La Cardio, Division of Cardiology, Bogota, Colombia; Department of Cardiovascular Medicine, Heart, Vascular, and Thoracic Institute, 9500 Euclid Ave., Cleveland Clinic, Cleveland, OH 44195, USA; Department of Cardiovascular Medicine, Heart, Vascular, and Thoracic Institute, 9500 Euclid Ave., Cleveland Clinic, Cleveland, OH 44195, USA

**Keywords:** cardioneuroablation, Vasovagal syncope, Intracardiac echocardiography, Epicardial fat pads

## Abstract

**Aims:**

Anatomical studies have documented a close topographical relationship between the ganglionated plexi (GP) containing parasympathetic inputs to the sinus node (SN) and atrioventricular node (AVN) and the epicardial fat pads (FPs) within the Waterston’s interatrial groove. We aimed to investigate the feasibility and outcomes of a novel anatomical approach to cardioneuroablation (CNA) that targets the atrial areas adjacent to the interatrial FPs identified with intracardiac echocardiography (ICE).

**Methods and results:**

About 17 patients [37.3 ± 10.2 years, 47% female] undergoing CNA for recurrent vasovagal syncope and documented sinus pauses (*n* = 13, 76%) and/or AVN block (AVB, *n* = 4, 16%) were included. The right superior RS-FP containing the RS-GP (target for SN vagal denervation) and the right inferior RI-FP containing the RI-GP (target for AVN vagal denervation) were identified with ICE and reconstructed on a 3D electroanatomic map. At baseline, all patients had provocable sinus pauses/AVB with extracardiac high-frequency vagal stimulation (ECVS). The target FPs could be identified in all patients and were adjacent to septal LA and RA sites covering an average surface area of 3.7 ± 1.4 cm^2^ and 2.97 ± 1.21 cm^2^, respectively. A total of 33 ± 15 RF ablations (30–40W, 60 s) were delivered to cover the target LA/RA area. A > 25% shortening of the PP interval was observed within the first 1–2 RF lesions in all cases. After ablation, complete abolition of sinus pauses/AVB response with ECVS was achieved in all patients, and 2 mg of atropine infusion resulted in no PP/PR interval change. After a median follow-up of 12 months (range 4–25 months), 16 patients (94%) remained free of recurrent symptoms (1 patient underwent repeat CNA for recurrent pre-syncope and AVB, 1 patient underwent PPM implant following ECG recording of asymptomatic diurnal AVB).

**Conclusion:**

An ICE-guided anatomical approach to CNA targeting visible FPs at the Waterston’s groove is a feasible and effective strategy to achieve SN/AVN vagal denervation, with good outcomes at mid-term follow-up.

## Graphical Abstract

**Graphical Abstract:**
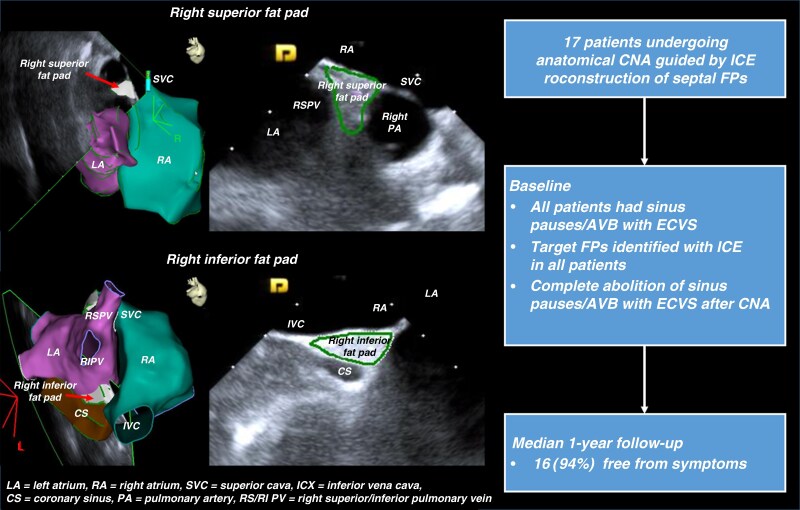

What’s New?

- Anatomical studies have documented a close topographical relationship between the ganglionated plexi (GP) containing parasympathetic inputs to the sinus node (SN) and atrioventricular node (AVN) and the epicardial fat pads (FPs) within the Waterston’s interatrial groove.
- This study investigated the feasibility and outcomes of a novel anatomical approach to cardioneuroablation (CNA) that targets the areas adjacent to the interatrial FPs identified with intracardiac echocardiography (ICE), and documented 94% freedom from recurrent symptoms after CNA at a median follow-up of 1 year.
- This technique may be associated with more reproducible identification of CNA targets. A prospective comparative study with other standard CNA techniques is needed

## Introduction

Vasovagal syncope (VVS) is the most common type of syncope across all age groups.^1^ The pathophysiologic mechanisms underlying VVS are complex and still incompletely understood, but they ultimately result in a reflex vagal response associated with bradycardia (i.e. cardioinhibition) and/or vasodilatation with hypotension and cerebral hypoperfusion. Pachon et al. first described a catheter-based ablation technique to treat patients with VVS and clinical evidence of cardioinhibition.^2,3^ The approach consisted of focal radiofrequency ablation of the left and right atrial tissue adjacent to the epicardial parasympathetic ganglionated plexi (GP), thus resulting in cardiac parasympathetic denervation and abolition of VVS episodes. Following this initial report, other investigators have documented similar benefits with cardioneuroablation (CNA) for VVS, with event-free survival during follow-up ranging from 71% to 100%.^3–6^ However, identification of the target atrial regions adjacent to the epicardial GPs remains difficult. Published studies have reported a variety of techniques to identify the target areas, ranging from intracardiac high-frequency stimulation to elicit parasympathetic responses (limited by proarrhythmic potential and lack of reliable GP stimulation with available tools),^2,3^ to analysis of fragmented atrial bipolar electrograms which may indicate proximity to the epicardial GP regions (limited by lack of specificity, sampling error and heterogeneous definition of electrogram fragmentation),^7^ and anatomical approaches targeting the putative atrial sites more likely to be adjacent to the GPs (limited by lack of direct visualization of the target epicardial areas to guide anatomical ablation).^8,9^

Anatomical and surgical studies have documented that the FPs within the Waterston’s groove contain the highest density of epicardial GPs providing parasympathetic innervation to the sinus node (SN) and atrioventricular node (AVN).^10–12^ Specifically, the right superior fat pad (RS-FP) in between the superior vena cava (SVC) and the septal aspect of the right superior pulmonary vein (RSPV) houses the largest concentration of GP supplying the SN,^13,14^ whereas the main GP area providing parasympathetic innervation to the AVN (right inferior fat pad, RI-FP) is located in the pyramidal space between the coronary sinus (CS) ostium, and the septal aspects of the inferior vena cava (IVC) at its junction with the right atrium (RA), and the right inferior pulmonary vein (RIPV).

In the present study, we evaluated the feasibility of direct imaging of the Waterston’s groove target FPs with intracardiac echocardiography (ICE) and reported the outcomes of a novel anatomical CNA approach targeting the left and right atrial regions adjacent to the FPs identified with ICE.

## Methods

We included consecutive patients with recurrent (≥2 episodes in the prior year) VVS refractory to first-line measures, ECG documentation of cardioinhibition (i.e. sinus pauses and/or AVN block correlated with the syncope events), and who underwent CNA guided by ICE visualization of the target FPs within the Waterston’s groove between October 2022 and July 2024 at two institutions (Cleveland Clinic, Cleveland, United States, and Fundación Cardioinfantil, Bogota, Colombia).

All patients were initially referred for a pacemaker implantation, and CNA was offered as a potential alternative to a pacemaker. All patients had a structurally normal heart per transthoracic echocardiography, normal 12-lead electrocardiogram and normal chronotropic function (based on analysis from extended ECG recordings). A baseline electrophysiologic study excluded abnormal SN function or infra-nodal conduction disturbances. Data were included in a prospective registry and approved by the IRB of the participating institutions for retrospective review.

### Intracardiac echocardiography identification and anatomic reconstruction of the target fat pads

**Figure 1:**
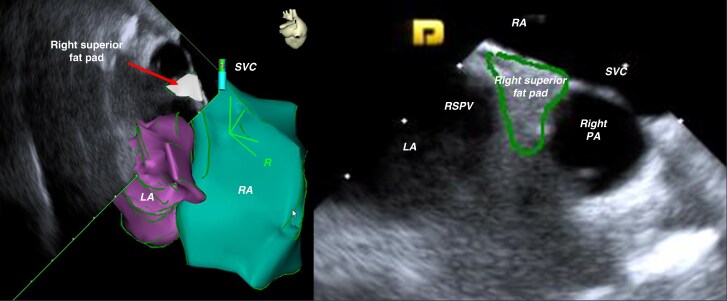
ICE visualization and reconstruction of the right superior fat pad (RS-FP). Note the topographic anatomical relationships visualized on ICE: the target RS-FP is contained in between the superior vena cava (SVC), the septal aspect of the right superior pulmonary vein (RSPV) and the lower border of the right pulmonary artery (PA).

**Figure 2:**
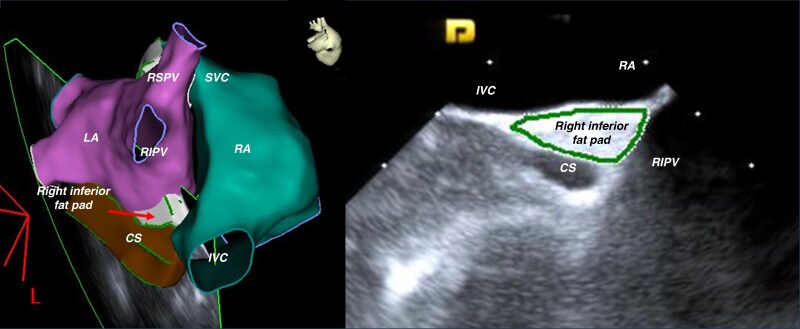
ICE visualization and reconstruction of the right inferior fat pad (RI-FP). Note the topographic anatomical relationships visualized on ICE: the target RI-FP is is located in the pyramidal space between the coronary sinus (CS) ostium, the septal aspects of the inferior vena cava (IVC) at its junction with the right atrium (RA), and the most anterior aspect of the right inferior pulmonary vein (RIPV).

**Figure 3:**
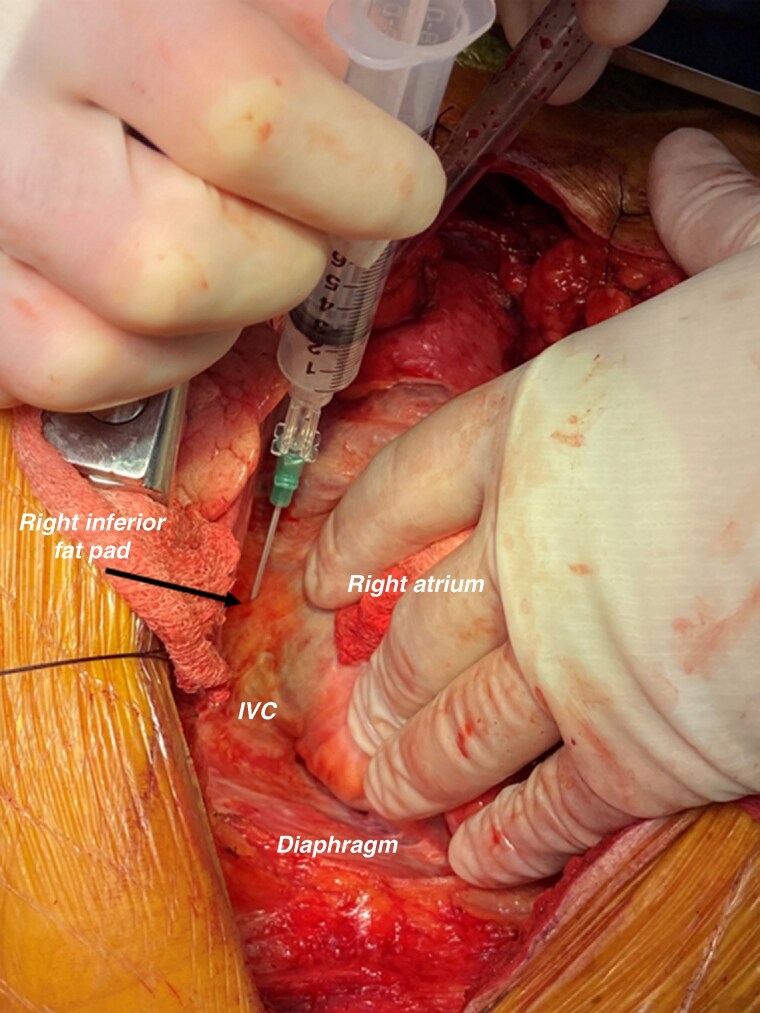
Intraoperative visualization of the RI-FP. The posterior-septal right atrium and the inferior vena cava are visualized. The left atrium and right inferior pulmonary vein are not in view.

A 64-element phased-array ICE catheter (AcuNav CARTOSound^TM^, Acuson, Mountain View, CA) was positioned within the right atrium. The RS-FP within the Waterston’s groove, which contains most of the GPs innervating the SN, was imaged by advancing the ICE probe to the junction between the SVC and RA and applying clockwise torque to image the septal SVC, the right pulmonary artery (PA) and the septal aspect of the RSPV (*Figure 1* and Supplementary material online, *Video S1*). The RS-FP could be visualised as a distinct hyperchoic structure between the septal RSPV, the lower border of the right PA and the septal SVC. The right inferior fat pad (RI-FP) within the Waterston’s groove, which contains most of the GPs innervating the AVN, was imaged pulling back the ICE catheter at the level of the CS ostium and applying clockwise torque to image the IVC-RA junction, the CS ostium and the inferior border of the RIPV (*Figure 2* and Supplementary material online, *Video S2*). Similar to the RS-FP, the RI-FP also appeared as a distinct hyperchoic structure contained between the roof of the CS ostium, the IVC-RA junction, and the inferior-septal border of the RIPV. Further validation of the topographic anatomical relationship between the target FPs within the Waterston’s groove and the adjacent RA and LA anatomical structures, as described above, was performed in two patients with structurally normal hearts undergoing surgical ablation of atrial fibrillation (*Figure 3*).

**Figure 4:**
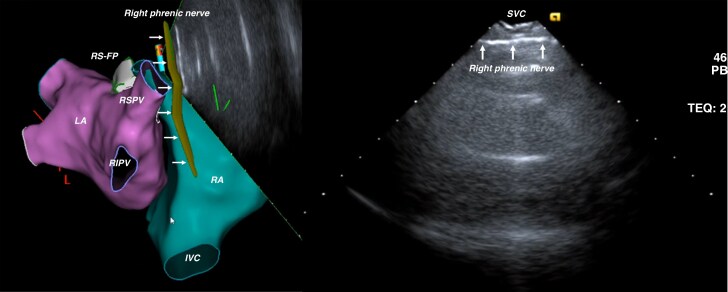
ICE visualization and reconstruction of the right phrenic nerve. The phrenic nerve can be imaged advancing the ICE probe to the superior vena cava (SVC) and rotating it to image the postero-lateral aspect of the SVC. Note how the phrenic nerve appears as hyperchoic linear structure (white arrows) adjacent to the outer surface of postero-lateral SVC.

The RS-FP and RI-FP were reconstructed and displayed within the three-dimensional electroanatomic map with the CartoSound^TM^ software (Biosense Webster, Irvine, CA) and used as an anatomical target for adjacent endocardial LA and RA ablation. Additionally, the right phrenic nerve location was reconstructed with ICE and imported within the electroanatomic map: The phrenic nerve appeared as a hyperchoic linear structure in the posterior-lateral RA (*Figure 4* and Supplementary material online, *Video S3*). A biatrial anatomical map was created with the CartoSound^TM^ software or with a multipolar catheter utilising the fast anatomical mapping (FAM) module (Biosense Webster, Irvine, CA).

### Cardioneuroablation procedure

Procedures were performed under general anaesthesia. Intravenous heparin was administered to target an activated clotting time >350 s. A multipolar (quadripolar or decapolar) catheter was advanced to the right (cases with predominant SN arrest) and/or left (cases with predominant AVN block) internal jugular vein up to the level of the maxillary dental arch and oriented medially to achieve extracardiac vagal stimulation (ECVS), as described by Pachon and colleagues.^15^ The catheter was connected to a commercially available nerve stimulator (SunStim^TM^ Plus, Sunmed) using a frequency of 50 Hz and an amplitude of 70 mA.^15^

**Figure 5:**
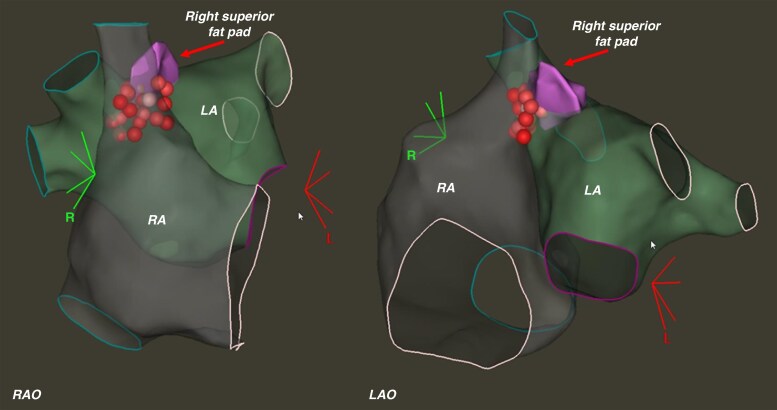
Representative example of biatrial right superior fat pad (RS-FP) ablation (purple structure in the map reconstruction) in a patient with recurrent vaso-vagal syncope and sinus arrest. RAO = right anterior oblique; LAO = left anterior oblique; LA = left atrium; RA = right atrium.

Catheter ablation was performed sequentially, starting from the LA (via transseptal access) and proceeding to the opposite RA regions adjacent to the reconstructed RS-FP (for patients with SN arrest, *Figure 5*) and RI-FP (for patients with AV block) with an open-irrigated contact force-sensing catheter (ThermoCool SmartTouch SF, Biosense Webster, Irvine, CA) utilising a power of 30–40W and point ablation duration of 60 s. Ablation targeted a minimum impedance drop of 10% from baseline values, and impedance trend was carefully monitored during the ablation to minimize the risk of steam pops. No other metrics were used to guide ablation. Ablation was not performed at LA/RA areas adjacent to the right phrenic nerve, as validated by ICE (see above) as well as high-output pacing (20 mA amplitude, 2 ms duration). No additional lesions were delivered within the LA and RA.

At the end of the ablation, which corresponded to a complete anatomical coverage of the RA/LA sites adjacent to the FPs reconstructed by ICE with a cluster of ablation lesions, ECVS was repeated to validate abolition of vagal responses, which was the ultimate endpoint of the procedure. Finally, 2 mg of atropine was infused to further demonstrate effective SN and AVN parasympathetic denervation, which was defined as no shortening in SN rate (i.e. PP interval on the surface ECG) or AVN conduction (i.e. PR interval on the surface ECG). Pre-procedural atropine challenge was not systematically performed.

### Follow-up

Post procedure, patients remained overnight in the hospital under continuous ECG monitoring. Beyond that, patients were followed up in the outpatient clinic. Information about any recurrent symptoms and SN pauses/AVN block was collected with two-week extended ECG monitors or implantable loop recorders (ILR) at 3, 6, and 12 months, and every year afterwards.

### Statistical analysis

Continuous data are reported as mean ± SD or median (25th to 75th percentile) for skewed distributions. Categorical data are reported as numbers and percentages. Statistical analyses were conducted by using Stata version 18 (Stata Corporation, College Station, Texas).

## Results

A total of 17 patients [37.3 ± 10.2 years, 8 (47%) female] underwent CNA (15 at the Cleveland Clinic and 2 at the Fundación Cardioinfantil) during the study period (*Tables 1* and *2*). The average body mass index (BMI) was 28.37 ± 8.31 kg/m^2^. Two patients had obstructive sleep apnea (OSA) at baseline (both treated with a continuous positive airway pressure machine). As mentioned, all patients had a history of recurrent syncope with ≥2 episodes in the prior year, and 24% of patients had ≥5 episodes. All patients had an ECG documentation of bradyarrhythmia correlating with the syncope episodes, including sinus pauses (*n* = 13, 76%) and/or AVN block (*n* = 4, 16%).

**Table 1:** Clinical characteristics of study patients

Patient #	Age, years	Sex	BMI (kg/m^2^)	OSA	ILR	Other ECG Documentation	Sinus Arrest	AVN Block
1	54	M	39.4	Yes	Yes	N/A	Yes	No
2	43	F	38.2	No	No	Holter	Yes	No
3	41	M	33	Yes	No	Telemetry	Yes	No
4	28	F	34.3	No	No	Zio Patch	No	Yes
5	28	F	24.2	No	Yes	N/A	Yes	No
6	18	F	20.9	No	Yes	N/A	Yes	No
7	38	M	28	No	No	Zio Patch	No	Yes
8	32	F	31.7	No	Yes	N/A	Yes	No
9	46	M	23.9	No	No	Telemetry	Yes	No
10	43	F	48.5	No	No	Telemetry	Yes	No
11	36	M	26.3	No	No	Telemetry	No	Yes
12	25	M	21.2	No	No	Telemetry	Yes	No
13	50	M	27.2	No	Yes	N/A	Yes	No
14	56	F	25.6	No	No	Telemetry	Yes	No
15	38	M	17.7	No	No	Holter	Yes	No
16	44	M	25.4	No	No	Tilt/Holter	Yes	No
17	24	F	16.8	No	No	Telemetry	No	Yes

**Table 2:** Main procedural findings and outcomes

Patient #	Procedure Duration, min	RF time, min	RS-FP Targeted	RI-FP Targeted	PP Interval, ms Baseline/End	PR Interval, ms Baseline/End	Follow-Up, months	Symptoms Recurrence
1	169	36.8	Yes	No	932/720	222/186	9	No
2	179	93.8	Yes	No	1504/1056	180/192	7	No
3	164	34.4	Yes	No	712/696	152/144	25	No
4	141	30.6	Yes	Yes	944/904	162/140	18	No
5	108	28.3	Yes	No	936/576	130/130	4	No
6	189	28.9	Yes	No	860/584	112/146	18	No
7	227	39	Yes	Yes	1128/720	156/146	13	No
8	153	17.6	Yes	No	688/512	122/128	6	Yes
9	130	29.1	Yes	No	1200/928	168/172	15	No
10	103	22	Yes	No	1608/728	194/190	12	No
11	136	39.8	Yes	Yes	1400/776	176/164	5	No
12	143	26.9	Yes	No	620/560	130/151	5	No
13	131	18	Yes	No	960/600	156/199	2	No
14	93	16.7	Yes	No	936/660	178/155	1	No
15	224	3.2	Yes	No	1250/650	160/150	6	No
16	240	19.1	Yes	No	1080/590	120/95	1	No
17	151	22.5	Yes	Yes	1000/720	180/171	3	No

At baseline, ECVS was able to reproduce SN arrest or AVN block in all cases (see Supplementary material online, *Figure S1*). The baseline PP interval was 1044 ± 278 ms, and the PR interval was 158 ± 29 ms. The target FPs could be identified in all patients with ICE and successfully reconstructed within the electroanatomic map. The mean time to complete mapping of the target FPs was 7 ± 5 min. The target FPs were adjacent to septal LA and RA sites covering an average surface area of 3.7 ± 1.4 cm^2^ and 2.97 ± 1.21 cm^2^, respectively.

The right phrenic nerve was also visualised with ICE in all cases and reconstructed within the electroanatomic map. High-output pacing demonstrated a perfect anatomical correlation between the areas of phrenic nerve capture and regions adjacent to the reconstructed right phrenic nerve.

Catheter ablation was focused on the RS-FP in patients with pre-procedural evidence of SN arrest and extended to the RI-FP if patients had clinically documented AVN block. A total of 33 ± 15 RF lesions were delivered to sequentially cover the target LA and RA areas. A > 25% shortening of the PP interval was observed within the first 1–2 lesions in all cases (see Supplementary material online, *Video S4*). At the end of the procedure, the PP interval was 704 ± 145 ms (*P* < 0.001 for comparison with baseline). For patients in whom the RI-FP was also targeted, the baseline and post-procedure PR interval were 160 ± 19 ms and 150 ± 16 ms, respectively (*P* = 0.12 for comparison). In all patients, repeat ECVS did not result in any change in PP interval or PR interval, and 2 mg of atropine infusion also resulted in no PP/PR interval change. No periprocedural complications were observed. The total RF time was 27 ± 10 min, and the total procedure time was 157 ± 43 min.

After a median follow-up of 12 months (interquartile range 4 to 25 months), 16 patients remained free from recurrent symptoms. One patient (monitored with ILR) who initially presented with syncope and SN arrest had recurrent pre-syncope and evidence of AVN block pauses (maximum 3.1 s) with no change in PP interval (see Supplementary material online, *Figure S2*). This patient underwent repeat ablation targeting the RI-FP with no further recurrences of symptoms at 10 months of follow-up after the last procedure. Another patient who initially presented with symptomatic vagally mediated high-grade AV block had evidence of recurrent asymptomatic diurnal AVB (maximum 4 s pause) on repeat Holter monitoring 1 month after the procedure. After discussion of the findings with the patient, a leadless pacemaker was implanted. Device checks revealed <1% RV pacing burden at 17 months follow-up. Another patient (patient #6, *Table 1*) had new-onset paroxysmal atrial fibrillation (AF) 1 year after the index CNA procedure and underwent AF catheter ablation (pulmonary vein isolation). Finally, two patients experienced symptomatic sinus tachycardia on follow-up, one managed conservatively and the other required a small dose of atenolol.

## Discussion

This study describes the outcomes of a novel anatomical CNA technique that targets the LA and RA areas anatomically adjacent to the Waterston’s groove FPs identified by ICE. The main findings are as follows:

1. Identification of the target FPs within the Waterston’s groove with ICE was possible in all patients. FPs identified on ICE could be efficiently (mean 7 min) reconstructed within the electroanatomic map and targeted from anatomically adjacent discrete LA and RA regions.
2. The reliability of the FPs reconstructed by ICE as an anatomical target for CNA was corroborated by a significant increase in heart rate within the first 1–2 RF lesions from the targeted sites, and further confirmed by a complete abolition of vagal responses at ECVS post-ablation and shortening of the PP/PR interval with atropine.
3. The clinical outcomes with the ICE-guided anatomical approach described in this report appear good, with elimination of recurrent symptoms in most patients at mid-term follow-up.

In patients with vasovagal syncope and predominant cardioinhibitory response, CNA procedures are being increasingly utilised worldwide owing to the promising results reported in single- and multicenter observational studies as well as small randomised controlled trials.^3,4,6,7,16,17^ One of the main challenges with CNA is an accurate and reproducible identification of the target atrial sites adjacent to the vagal GPs. In this regard, studies have reported a variety of indirect techniques for GP localization ranging from anatomical approaches based on electroanatomic reconstruction of the right and left atrial geometry and empirical ablation of anatomical sites putatively adjacent to the target GPs, to ablation of atrial areas where vagal responses can be elicited with local high-frequency stimulation and/or sites displaying multicomponent fractionated bipolar electrograms that are thought to indicate myocardial uncoupling with interpolated visceral neurons adjacent to vagal GPs.^3,4,6,8,16^

In order to overcome the limitations inherent to the indirect and nonspecific GP site identification with the aforementioned techniques, recent studies have investigated the feasibility of pre-procedural contrast-enhanced computed tomography (CT) to directly visualise epicardial FPs where vagal GPs are anatomically embedded.^17–19^ Interestingly, Benabou et al. have reported a poor correlation between atrial sites displaying multicomponent fractionated electrograms and the location of epicardial FPs detected at CT, thus suggesting that published indirect approaches to identify target atrial sites based on EGM analysis are limited by poor sensitivity and specificity.^19^ Early clinical experiences with epicardial FPs, CT imaging integration within the electroanatomic mapping system as an anatomical target for CNA are encouraging.^17,18^

Notwithstanding the high anatomical accuracy, CNA guided by pre-acquired CT reconstruction of the epicardial FPs has some important limitations, which include obligatory patient exposure to relatively high-dose radiation (particularly relevant in the young patient population referred for CNA procedures) and registration inaccuracy with real-time mapping system data. The approach described in the present investigation resolves these issues as it relies entirely on ICE imaging of the FPs, which is a non-fluoroscopic imaging modality, and also accurately integrates in real-time the reconstructed FPs within the electroanatomic mapping system with dedicated ICE integration modules. As mentioned, the anatomical accuracy and topographic relationships of the target FPs identified with ICE were validated in two patients with structurally normal hearts undergoing surgical ablation of atrial fibrillation and, most importantly, demonstrated by the effect observed with RF application at the adjacent atrial sites resulting in a nearly immediate increase in heart rate, abolition of ECVS/atropine responses and favourable follow-up outcomes. In this context, it is possible that the use of general anesthesia may have affected the baseline parasympathetic tone, but this should not substantially impact the cardioinhibitory responses observed with ECVS. As ECVS employs high-voltage and high-frequency stimulation at the level of the upper neck, which typically also elicits significant neck muscle contractions/spasms with stimulation, all procedures were performed under general anaesthesia for patient comfort. Of note, in all cases, ECVS was able to elicit a cardioinhibitory response at baseline, which was successfully eliminated following CNA, despite no change in anaesthesia protocols/agents during the procedure. We consider this adequate proof of successful acute parasympathetic denervation, although the optimal procedural approaches and endpoint(s) to establish effective vagal denervation are still a matter of debate, and to date, there has not been a proper multicenter prospective head-to-head comparison of different acute procedural endpoints for CNA.^20,21^

In our study, we employed selective ablation of the septal FPs, which correspond to the septal GPs. This more focused approach has been utilised before with comparable outcomes to more extensive ablation approaches, including also the lateral LA GPs (adjacent to the left superior and inferior PVs).^2,8,9,22,23^ From a mechanistic perspective, non-septal LA GPs connect to the SN and AVN via the septal GPs, which explains why focal ablation of the septal GPs can be sufficient to achieve the desired denervation effect.^23^

After a median of 12 months from the CNA procedure, 16 out of 17 patients were asymptomatic. In two cases, recurrent AV block (symptomatic in 1 case) was documented on post-ablation ECG monitoring. In one case, the initial vasovagal syncope cardioinhibitory presentation was SN arrest, and the index ablation focused only on the RS-FP. During follow-up, recurrent pre-syncope episodes were associated with AV block and no change in PP interval (see Supplementary material online, *Figure S2*), suggesting durable SN vagal denervation but persistent cardioinhibition of the AVN. As mentioned, this case was managed with an additional CNA procedure targeting the RI-FP, with no further recurrences at 10 months of follow-up. The second patient opted to receive a leadless PPM (a repeat CNA procedure was also discussed), given the ECG evidence of recurrent diurnal AVB. Of interest, one patient (patient #6: 18-year-old female with recurrent vasovagal syncope and SN arrest) developed recurrent paroxysmal AF 1 year following the index CNA procedure targeting the RS-FP. Although the occurrence of AF in this case could have been coincidental, we cannot rule out pro-arrhythmia from unopposed sympathetic atrial innervation (which may mimic AF trigger induction with adrenergic drug agents such as isoproterenol), a potential adverse effect of CNA that has not been documented previously.^6,23^

Finally, ICE also allowed accurate identification of anatomical structures, such as the right phrenic nerve, that should be preserved during CNA procedures. High-output pacing demonstrated a perfect anatomical correlation between the areas of phrenic nerve capture and regions adjacent to the reconstructed right phrenic nerve. This finding is of interest and, if confirmed in larger studies, can have implications beyond CNA procedures (e.g. pulmonary vein isolation or ablation of atrial tachycardias adjacent to the phrenic nerve).

## Limitations

The main limitations of this study are inherent to its observational design and highly selected cohort of patients with recurrent vasovagal syncope episodes and documented cardioinhibition who were initially referred to our centres for PPM implantation. The study sample size was relatively small and, although outcome results were excellent at a median follow-up of 12 months, longer-term follow-up data are necessary to evaluate the durability and safety of this novel CNA approach. As mentioned, the described technique relies exclusively on intraprocedural ICE imaging. As such, our results may not be generalised to centres that do not routinely use ICE for complex ablation procedures or operators who are not proficient with ICE imaging. Finally, other techniques for identification of the target sites, such as analysis of fractionated electrograms and/or intracardiac high-frequency stimulation, were not adopted in this study. In our experience, induction of AF is common with atrial high-frequency stimulation. As most patients included in our series had sinus arrest as the predominant clinical manifestation of cardioinhibition with their vasovagal syncope episodes, induction of AF with high-frequency atrial stimulation would have limited the assessment of sinus arrest response. Similarly, systematic addition of the atropine test pre-procedure would have been helpful to compare with the acute response to atropine observed after CNA. A properly designed prospective study comparing our approach with the standard electrogram-guided technique with atrial high-frequency stimulation is needed to conclusively demonstrate whether our technique offers advantages over a standard CNA approach.

## Conclusions

An ICE-guided CNA anatomical approach that identifies and reconstructs the target FPs within the Waterston’s groove is feasible and is associated with good acute and mid-term follow-up outcomes. Larger studies with longer follow-up duration are needed to determine the durability and safety of the vagal denervation effect achieved with this technique.

## Supplementary Material

euaf295_Supplementary_Data

## Data Availability

The data underlying this article will be shared on reasonable request to the corresponding author.
