# Supplementary figures and images for: Intracardiac echocardiography guided anatomical approach to cardioneuroablation: feasibility and outcomes
Source: Europace. 2025 Nov 18;27(12):euaf295. doi: 10.1093/europace/euaf295 (PMC13223727; doi:10.1093/europace/euaf295)

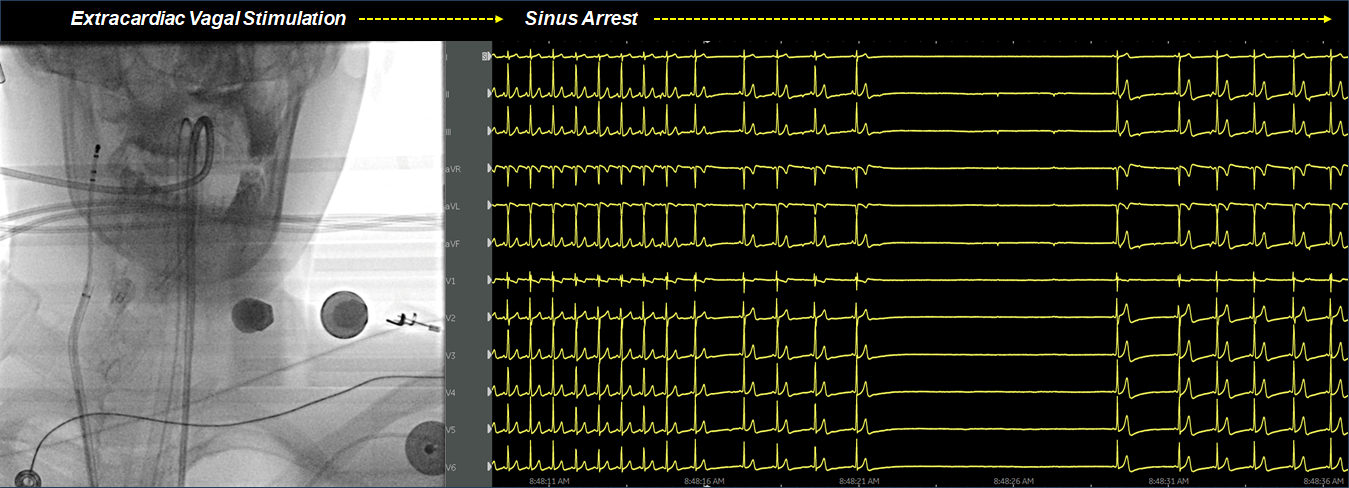

Supplement: euaf295_Supplementary_Data [file euaf295_supplementary_data.zip › Supplemental_Figure_1.tif]

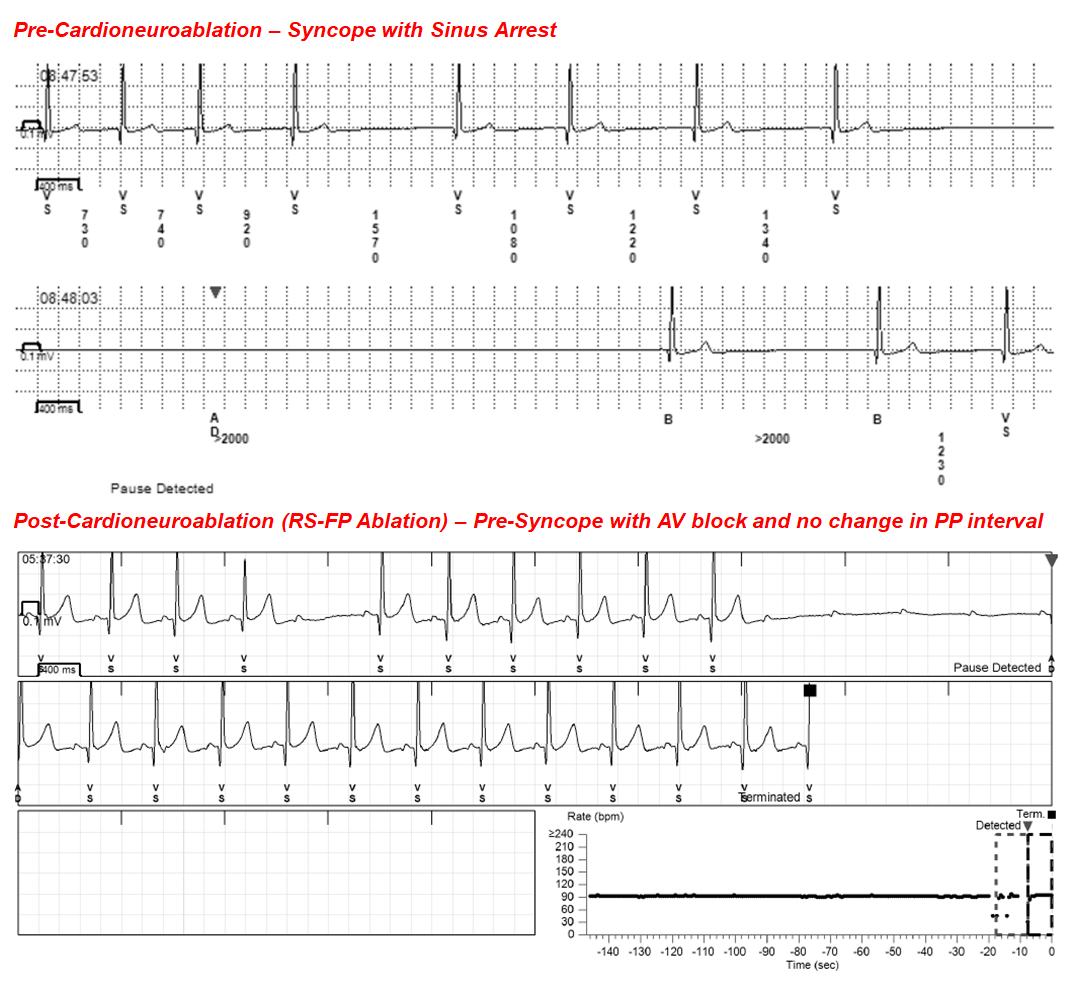

Supplement: euaf295_Supplementary_Data [file euaf295_supplementary_data.zip › Supplemental_Figure_2.tif]
